# The Pharmacoresistant Epilepsy: An Overview on Existant and New Emerging Therapies

**DOI:** 10.3389/fneur.2021.674483

**Published:** 2021-06-22

**Authors:** Antonella Fattorusso, Sara Matricardi, Elisabetta Mencaroni, Giovanni Battista Dell'Isola, Giuseppe Di Cara, Pasquale Striano, Alberto Verrotti

**Affiliations:** ^1^Department of Medicine and Surgery, Pediatric Clinic, University of Perugia, Perugia, Italy; ^2^Child Neurology and Psychiatry Unit, Children's Hospital “G. Salesi”, Ospedali Riuniti Ancona, Ancona, Italy; ^3^Pediatric Neurology and Muscular Diseases Unit, IRCCS “G. Gaslini” Institute, Genoa, Italy; ^4^Department of Neurosciences, Rehabilitation, Ophthalmology, Genetics, Maternal and Child Health, University of Genoa, Genoa, Italy

**Keywords:** drug resistant epilepsy, pharmacoresistance, antiseizure medications (ASMs), rational polytherapy, fenfluramine, cannabidiol

## Abstract

Epilepsy is one of the most common neurological chronic disorders, with an estimated prevalence of 0. 5 – 1%. Currently, treatment options for epilepsy are predominantly based on the administration of symptomatic therapy. Most patients are able to achieve seizure freedom by the first two appropriate drug trials. Thus, patients who cannot reach a satisfactory response after that are defined as pharmacoresistant. However, despite the availability of more than 20 antiseizure medications (ASMs), about one-third of epilepsies remain drug-resistant. The heterogeneity of seizures and epilepsies, the coexistence of comorbidities, and the broad spectrum of efficacy, safety, and tolerability related to the ASMs, make the management of these patients actually challenging. In this review, we analyze the most relevant clinical and pathogenetic issues related to drug-resistant epilepsy, and then we discuss the current evidence about the use of available ASMs and the alternative non-pharmacological approaches.

## Introduction

Over the past 20 years, a significant number of antiepileptic drugs, more appropriately defined as antiseizure medications (ASMs), were developed and licensed. However, at least 30% of people with epilepsy have drug-resistant epilepsy (DRE), so they remain refractory to common pharmacological treatments (1). The International League Against Epilepsy (ILAE) refers to drug-resistant epilepsy as the failure of adequate trials of two tolerated, appropriately chosen, and used ASM schedules, whether as monotherapy or in combination, to achieve sustained seizure freedom (2). According to this definition, an observational study analyzed the response to ASMs in hospitalized patients with newly diagnosed epilepsy and showed that the first and second drug regimens were successful in 49.5 and 36%, respectively. In this regard, all the therapies with ASM after the failure of the first two drugs had a significantly lower rate of success (from 12.5 to 22.2%) (3). According to these data, the chances of controlling seizures seem to drastically decline after the failure of the second ASM. Some clinicians would avoid trying other pharmacological treatments in those patients who may befit from surgical treatment with a higher percentage of success (4). The concept of pharmacoresistance not only implies intractable seizures, but the underlying pathogenesis is responsible for structural and neurobiochemical changes that also cause cognitive and neuropsychiatric disturbances and psychosocial dysfunction (5). Due to the extreme variability of DRE patients phenotypes and the lack of a unique shared definition of ASM effectiveness in achieving seizure freedom, it remains challenging to compare clinical trials and define practice guidelines. However, according to ILAE, who tried to give an operational definition, an ASM is deemed effective if there is a seizure-free period of classically 12 months or of at least three times the longest pretreatment inter-seizure interval (2). Pharmacological therapy should be appropriately chosen for the epileptic syndrome and the type of seizures and administered for a minimum of 6 months at an adequate dosage (6). Optimal effective dose range and frequency of administration depend on individual response, ongoing comorbidities, and medication tolerability, with adverse side effects limiting further the choice of ASMs (7). Therefore, it is clear that patients with DRE represent a spectrum of different clinical and neurobiological pictures rather than a group of patients with the same disease, requiring a complex approach to several issues that we will further try to focus on.

The aim of this review is to outline an overview of the most relevant clinical and pathogenetic issues related to DRE, and examine a practical approach with ASMs, the potential of novel emerging ASMs, and alternative non-pharmacological therapies for patients with DRE.

## The Burden of Drug-Resistant Epilepsy

Despite the wide variation in DRE definition, a recent epidemiological systematic review reported an overall incidence proportion of DRE ranging from 0.06 to 0.51% and a prevalence ranging from 0.11 to 0.58%. Among the analyzed studies, the pooled estimate prevalence of DRE was 0.30 (95% CI: 0.19–0.42), consistent with what has been frequently reported in the literature. The pooled incidence proportion was 0.15 (95% CI: 0.11–0.19) in children, and 0.34% (95% CI: 0.06–0.62) in adults, with an overall pooled incidence of 20% (95% CI 0.14–0.27) (8).

Common causes of treatment failure are poor compliance and an inappropriate selection of ASM due to epileptic seizures misdiagnosis. A trust based relationship with patients and their families is mandatory to ensure a good understanding of the problem and optimal adherence to the prescribed drug regimen. On the other hand, a “false pharmacoresistance” should be ruled out in any patient presenting difficult-to-treat seizures. Causes of apparent pharmacoresistant epilepsy are the misdiagnosis of psychogenic non-epileptic events as seizures and the inability to identify the correct type of epilepsy leading to inappropriate drug selection. Other issues that could lead to “false pharmacoresistance” are the prescription of inadequate drug dosage and incorrect assessment of the response to treatment with overdiagnosis of side effects (i.e., drug interactions leading to increased side effects and poor tolerability) (9).

As a further complication, even patients correctly labeled as pharmacoresistant can have phases of long complete remission (pharmacoresponsive) alternating with a relapsing course (pharmacoresistant). However, in a study cohort of adults with DRE, among patients with a 12-months seizure remission period, the risk of seizure relapse remained high (71.2% at 5 years). Therefore, it is reasonable to be cautious when discussing the likelihood of stable remission (10).

It is noteworthy that people with DRE have from 2 to 10 times higher risk of sudden death compared to the general population. The sudden unexpected death in epilepsy (SUDEP) is defined as sudden, unexpected death of someone with epilepsy who is otherwise healthy, in which no other causes of death may be found when an autopsy is done. The death often occurs during the night, and it can be witnessed or not, without necessary evidence of a recent convulsive seizure (11). The risk of SUDEP is inversely related to seizure control, especially in patients with a higher rate of convulsive seizures and long-standing epilepsy (12, 13). The causes of this epilepsy-related death have been extensively analyzed in several studies, but definite predictive factors of SUDEP are still lacking. In this regard, the only way to prevent this complication in pharmacoresistant patients is to optimize seizure control.

Besides SUDEP and other causes of premature death in people with DRE (e.g., seizure-related accidents, cerebral neoplasms, and neurodegenerative diseases underlying symptomatic epilepsies), many different comorbidities may affect these patients. Cognitive and neuropsychiatric disorders such as depression and anxiety in adults, attention deficit with hyperactivity disorder, autism spectrum disorders, and neurobehavioral problems in children are more common than in the general population (14, 15). These comorbidities worsen language and social skills, adversely affecting long-term psychosocial functioning. The spectrum of disabilities can vary broadly, resulting from underlying structural, functioning, or genetic etiopathology that has led to seizures or the negative effect of ASM therapies. In rare selected cases, a targeted therapy on the underlying cause of epilepsy can ameliorate both seizures and cognitive dysfunctions, as the ketogenic diet for GLUT1 deficiency syndrome. In many epileptic encephalopathies, prompt initiation of an effective ASM therapy may improve cognitive and neurobehavioral symptoms (16). However, these comorbidities could persist despite an adequate ASM therapy with seizure freedom, maybe due to synaptic reorganization or impaired neurogenesis, and even more represent an adjunctive management issue in DRE patients. Furthermore, other diseases such as migraine, cardiovascular disorder, asthma, osteoarthritis, and gastroesophageal reflux have a higher incidence in people with epilepsy. The mechanisms of association identified vary from casual, causative, resultant, bidirectional, or shared risk factors. For instance, epilepsy can be caused by comorbidities like cerebrovascular diseases or perinatal stroke, or vice versa aspiration pneumonia, and seizure-related fracture can be considered resultant comorbidities of epilepsy. Sometimes the conditions share the same risk factors or the same genetic disorder responsible for both, or in other cases, the relationship is bidirectional as in autism spectrum disorders (17).

## Predictive Risk Factors

Discerning patients who are at higher risk of drug refractoriness could be helpful. Several studies have attempted to identify predictive factors for DRE development, but they all lack a unique shared definition of drug refractoriness and are affected by the heterogeneity of the examined population. Diagnosing certain types of epileptic syndromes in pediatric age already means giving a prognostic evaluation of drug resistance: Lennox-Gastaut syndrome (LGS), early infantile epileptic encephalopathy, Dravet Syndrome (DS), or Rasmussen encephalitis are almost pharmacoresistant, maybe due to the intrinsic neurobiological pattern underlying epilepsy (7). Several clinical factors have been associated with DRE, such as age at onset of epilepsy (<1 year), etiology, abnormal neuroimaging, the coexistence of neuropsychiatric disorders or intellectual disability, history of prolonged febrile seizures, or status epilepticus, and the presence of specific EEG abnormalities (18). Seizure onset during the neonatal period is more likely associated with a higher risk of developing DRE than epilepsy onset later in life. Idiopathic epilepsies have a lower risk of pharmacoresistance than symptomatic epilepsy, which means epilepsy with underlying structural abnormalities such as cortical dysplasia, mesial temporal sclerosis, tuberous sclerosis, or vascular lesions. Focal seizures are suggested to have a higher risk compared to generalized seizures (18). Gender is not regarded as a risk factor, while family history is still controversial according to different studies (9, 18). The number of seizures occurring in the year before starting treatment, previous history of drug abuses, and family history of epilepsy in first-degree relatives were positively associated with DRE, according to Chen and colleagues (18). Overall, in a cohort of patients with newly diagnosed epilepsy of all types, more than half of the patients became seizure-free during treatment with a first ASM, about 15% became seizure-free during treatment with a second or a third drug, while only 3% of epilepsy cases was controlled by treatment with two drugs. This suggests that an inadequate response to the initial ASM therapy is more likely associated with the development of refractory epilepsy (19). Furthermore, also time to achieving seizure freedom could be a prognostic value for long-term outcome in epileptic patients. According to a *post-hoc* analysis on patients treated for focal epilepsy, patients who were seizure-free at 6 months had a 90% chance of being seizure-free at 12 months, whereas those not seizure-free at 6 months had only a 45% chance of being seizure-free at 12 months, suggesting that the clinical response at 6 months was an excellent predictor of response at 12 months (20).

## Pathogenesis

The mechanisms underlying DRE are not completely known. The pathogenesis of drug resistance is likely variable and multifactorial, with several mechanisms acting together in a given patient (6). In this regard, besides clinical evidence, experimental models provide better characterization and understanding of the putative mechanisms of ASM failure. The hypothesized mechanisms of drug resistance may be intermingled and comprise disease-related mechanisms, drug-related mechanisms, and genetic mechanisms.

According to the “transporter hypothesis,” the increased expression or function of multidrug efflux proteins in human epileptic brain tissue and in animal models of DRE decreases the effectiveness of ASMs, irrespective of their target of action (21, 22). The ATP-dependent P-glycoprotein (P-gp) is the product of the human multidrug-resistance-1 (MDR1; ABCB1) gene, and due to its broad substrate specificity, it plays a role in restricting brain entry of multiple different drugs. This protein is located in brain capillary endothelial cells that form the blood-brain barrier (BBB) and acts together with other multidrug resistance proteins in order to protect the brain from intoxication by lipophilic xenobiotics that otherwise would cross the BBB by passive diffusion (22, 23). This mechanism is the basis of treatment failure of different brain tumors, brain infections, and several other brain disorders (22). Several ASMs show similar chemical structures to P-gp substrates, thus the increased expression of P-gp and other efflux pumps may limit their entry across the BBB, conferring a multidrug-resistant epilepsy phenotype (24). However, evidence supporting overexpression of P-gp in epileptogenic brain tissues is controversial, with the role of other multidrug resistance proteins still unknown (21). The overexpression of drug efflux proteins seems to be restricted to epileptic focus sparing adjacent normal tissue, explaining the absence of fewer neurotoxic side effects in ASMs refractory patients than responsive ones (25). Furthermore, whether the overexpression in DRE patients is intrinsic (genetic) or acquired as a consequence of uncontrolled seizures or chronic ASM treatment remains unclear. The experimental evidence supports the transported hypothesis since ASM non-responders show higher expression of P-gp at the BBB than responders in rat models. Moreover, overexpression of P-gp is associated with lower brain levels of ASMs in rodents, and above all, the inhibition of P-gp by tariquidar counteracts resistance to ASMs in a rat model of temporal lobe epilepsy (TLE) (11, 22). However, the transporter hypothesis is still controversial, and further studies are needed to assess better the clinical relevance of efflux transporters overexpression at the BBB.

The “pharmacokinetic hypothesis” suggests that the overexpression of efflux transporters is localized in the peripheral organs, such as liver, intestine, and kidneys, thus decreasing ASM plasma levels and the amount of ASM available to cross the BBB. In this regard, multidrug transporters expression is not necessarily restricted to the brain but may also occur in other organs and tissues. Animal studies do not support this hypothesis, and evidence for it is quite limited (11).

Alternatively, the “target hypothesis” postulates that acquired epilepsy-induced alterations to the structure or the functionality of ASMs target molecules lead to a reduction in their response to treatment (26). This theory is primarily based on studies with carbamazepine (CBZ) on voltage-dependent sodium channels in hippocampal neurons of patients with mesial TLE. The use-dependent block of voltage-sodium channels of dentate granule cells by CBZ was completely lost in patients with CBZ resistant epilepsy, compared with neurons from patients without mesial TLE. The same results were obtained blocking voltage-sodium channels with pilocarpine rat models for CBZ and phenytoin (PHT) (26, 27), but not with other ASMs such as lamotrigine (LTG) and valproic acid (VPA) (27). It still remains unclear if the loss of sodium channel sensitivity in CBZ resistant patients could be extended to other ASMs with similar mechanisms of action. Consistent with the target hypothesis, other sites of action like GABAa receptors have been studied. Alteration in sensitivity of GABAa receptors has been reported in animal models of DRE, but no clinical evidence supports it (9, 28). Therefore, even though the target hypothesis appears to be conceivable, the evidence so far available is still limited.

Furthermore, epilepsy may induce structural alterations that include neurodegeneration, axonal sprouting, synaptic reorganization, neurogenesis, and gliosis, which are the basis of the “neural network hypothesis” (29). These changes cause the formation of an abnormal neural network that would lead to ASMs resistance. Hippocampal sclerosis supports this theory; thus, it is thought to play a causal role in the onset of pharmacoresistance in TLE, while following surgical resection often reverses resistance (30). However, alterations in the neural network do not lead to refractoriness in all epileptic patients, suggesting the requirement of other causal factors acting together (24). Experimental evidence highlights the findings that hippocampal damage is related to ASM resistance in a rat model of DRE.

On the contrary, the “intrinsic severity hypothesis” regards pharmacoresistance as an inherent property of epilepsy-related to disease severity (31). High seizure frequency is a reliable factor of pharmacoresistance; however, it is not the only predictor of pharmacoresistance. Animal models exhibiting very high seizure frequency are usually non-responders, even though some of non-responders epileptic rats may also have low seizure frequency. Therefore, little evidence supports this hypothesis.

The “genetic hypothesis” identifies in gene single polymorphisms variants the reason for the different susceptibility to pharmacoresistance in patients with epilepsy (32). This hypothesis is based on the concept that there is an endogenous variation in people with epilepsy, leading to a reduced chance of controlling seizures with ASMs. Unfortunately, there are not yet generally accepted genetic associations to support the model of syndrome-independent mechanisms of drug resistance driven by genetic variation, since so far, the studies available are of limited size, involving different patients groups and only selected single polymorphisms variants (e.g., SCN1A, ABCB1) (33).

Neuroinflammation and BBB dysfunction may play an important role as potential mechanisms in promoting and sustaining epileptic activity (34–36). An enhanced BBB permeability is present in experimental models and clinical conditions. A BBB dysfunction is usually associated with a concomitant neuroinflammatory response in the same tissue areas. In all these conditions, there is a P-gp induction in brain vessels and astrocytes. Neuroinflammation and BBB dysfunction are, therefore, hallmarks of an epileptogenic zone in various forms of DRE and in animal models of acquired epilepsies (11). In this regard, preclinical studies in experimental models of acute seizures and chronic epilepsy showed that neuroinflammation in brain areas of seizure onset and generalization plays a pivotal role in neuronal hyperexcitability underlying seizure generation. Microglia and astrocytes are crucially involved in both the induction and perpetuation of the inflammatory response to epileptogenic injuries or seizures; other contributors are neurons, cell components of the BBB, and leukocytes (37). Furthermore, specific inflammatory molecules and pathways have been identified to influence outcomes in various experimental models of epilepsy (38). Growing evidence demonstrates that neuroinflammation, BBB changes in permeability, astrocytic dysfunction lead to epileptogenesis, epilepsy progression, and ASM resistance (11, 37, 38). Antibody-mediated encephalopathies are inflammatory brain diseases increasingly recognized as a cause of seizures and status epilepticus, which are refractory to conventional ASMs but may benefit from immunotherapy. An early diagnosis of these conditions leads to a tailored treatment with immunomodulatory agents, and, in most cases, symptomatic treatment with ASMs can be discontinued after the acute phase. The subsequent risk of developing chronic epilepsy is relatively low (10–15%) and may vary depending on the target antigen and prompt immunotherapy. Chronic epilepsy after antibody-mediated encephalopathies is usually characterized by drug-resistant seizures that may result from an ongoing inflammatory process that persists beyond the acute phase or as sequelae due to irreversible changes altering the neuronal networks and persisting after the inflammatory process resolves (34, 39).

## Pharmacological Therapy in Drug-Resistant Epilepsy

### Monotherapy vs. Rational Polytherapy at New ASMs Era

Despite recent advances in the field of epilepsy and the introduction of new ASMs over the past 20 years, the management of DRE remains complex and leaves many questions unanswered. Pharmacological therapy is the mainstay of refractory epilepsy management, and in this class of patients, polytherapy should be carefully evaluated, accounting of risk/benefit ratio in terms of efficacy and tolerability, as well as the compliance of the patient. The most important questions by physicians about choosing ASMs in patients with DRE are “how can I select patients with DRE candidate to polytherapy, and how can I conduct the optimal treatment regimen for them?.” The lack of proper evidence-based guidelines that may guide the physician in the choice of the most effective and appropriate drug regimens makes the management of DRE patients complicated. However, we further discuss theoretical recommendations that can guide the clinician to the so called “rational polytherapy” for DRE. This process necessitates systematic ASMs trials after the failure of at least two drug regimes properly tried.

First-generation ASMs were limited in numbers, mechanisms of action, pharmacokinetic (strong inducers or inhibitors), and tolerability profile due to a high rate of adverse events (AEs). Only one randomized controlled trial (RCT) comparing CBZ as monotherapy with combination therapy of CBZ and VPA as initial treatment regimen in patients with untreated generalized tonic-clonic and/or partial seizures was conducted: the outcomes were in favor of combination therapy even if not statistically significant, but this combination showed relevant pharmacokinetic drug interactions (40). Before being accepted on the market, new ASMs underwent rigorous RCTs in patients taking from one to three ASMs, showing the superiority of new ASMs to the placebo as add-on therapy. Evidence shows better pharmacological profiles, including linear pharmacokinetics, less potential for drug interactions, and different mechanisms of actions that may be combinate in the association therapy. After many years of use in polytherapy, new ASMs have been studied in comparative monotherapy RCTs. Some of them are now also indicated as first-line drugs for focal-onset seizures or specific types of epilepsy (41–43).

The aim of polytherapy in pharmacoresistant patients is to identify ASMs combinations that maximize efficacy and minimize side effects (40). There are limited human clinical studies on the best combinations of ASMs; therefore, the choice of a second or third drug in a rational polytherapy should also account for animal studies and empirical considerations (44). Experimental animal models allowed to assess pharmacodynamic interactions in combination therapy that are primarily related to the mechanisms of actions of ASMs, through isobolographic analysis or direct measurement of therapeutic index. Desirable effects of combination therapy should be an antiseizure supra-additive effect (synergy effect) and possibly neurotoxic antagonism or neurotoxic infra-additive effect (45, 46). Higher incidence of AEs and lower efficacy were detected in clinical trials that compared combinations of drugs with sodium-channel blocking effects, compared to combination therapy of a sodium-channel blocker and a drug with a different mechanism of action like a GABAergic drug (47–49). Hence, the hypothesis of ASMs synergistic interactions driven by mechanisms of action, according to which the choice of ASMs combination based on different mechanisms of action may increase the treatment effectiveness. In a large population-based study on patients with focal seizures, it emerged that ASMs regimens with different mechanisms of action had better results both in treatment duration and in the risk of hospitalization and admission to emergency departments (50).

The combination with the best evidence for synergism in human studies is VPA with LTG. The synergistic interaction between these ASMs was reported in several studies, which demonstrated a major response rate with the use of LTG as add-on therapy with VPA compared to the addition of LTG to CBZ or PHT (51). The association of VPA with ethosuximide (ETX) was regarded more effective than VPA or ETX as monotherapy in children with difficult to treat absence seizures (52). Other combination regimens studied in a cohort of patients with focal seizures are LTG-LEV (53) and lacosamide (LCM)-LEV (54, 55). The synergic effect of these ASMs combination may result from the association of different mechanisms of action. Indeed, LCM enhances voltage-gated sodium channels slow inactivation, while LEV modulates synaptic neurotransmitter release through binding the synaptic vesicle protein SV2A in the brain (53, 54). Association regimens of LTG-TPM and VPA-LEV in adults could be useful, although with a low level of evidence (56, 57). A good level of evidence subsists for the use of stiripentol (STP) as add-on therapy in combination with clobazam (CLB) and VPA in children and adolescents with DS. STP was approved as adjunctive therapy for DS in Europe in 2007, following in Japan (2012), Canada (2012), and the USA (2018) (58). Two controlled trials performed in 2000 (59) and 2002 (60) in young patients with DS who were already receiving VPA and CLB treatment showed a significantly higher response rate in those treated with STP than placebo. These results were confirmed by subsequent observational retrospective and prospective long-term studies in terms of seizure control, reduction of prolonged seizures, number of episodes of status epilepticus, and hospitalizations. The anti-seizure effects of STP observed in DS may result from two different mechanisms. One pharmacokinetic, which provides the increase of CLB active metabolites mediated by STP, and one regarding the mechanism of action of STP, is an enhancement of gamma-aminobutyric acidergic transmission through post-synaptic GABA receptors in a site of action different from benzodiazepines (58). Finally, other new ASMs such as eslicarbazepine acetate, gabapentin, and zonisamide have proven to be effective as additional drugs in the treatment of DRE (44). However, even with the increasing interest in new ASMs and their recommended use in DRE, none of them have proven their superiority over conventional ASMs in head-to-head comparison studies in terms of efficacy (61).

The other issues regarding the use of rational polytherapy are the different pharmacokinetic and AEs profiles and the consequences of drug-drug interactions. As mentioned before, older ASMs have many interactions with other drugs (ASM or others), mostly mediated by their effect on the cytochrome P450 as enzymatic inhibitors or inducers. For example, when considering the association between VPA and LTG, it is important to consider that VPA acts as a potent enzymatic metabolic inhibitor; thus, it can reduce the clearance of LTG, increasing its haematic level and, therefore, the probability of LTG-induced hypersensitivity or tremor development (62). In this contest, the physician should slowly titrate LTG, starting with lower doses of the latest (44). CBZ, phenobarbital (PB), and PHT are instead inducer-enzymes that can reduce the levels of anticoagulants, oral contraceptives, or immunosuppressants. Furthermore, these older ASMs have a spectrum of AEs that range from VPA-associated hepatotoxicity and encephalopathy to bone marrow suppression induced by CBZ, PHT, and PB (63). Conversely, new ASMs have fewer pharmacokinetic interactions (most of them are weak enzyme inducers or inhibitors) and better tolerability profiles; hence, they are optimal candidates for combination therapy (64). In this regard, LEV has the fewest pharmacological interactions, and together with TPM and zonisamide is widely used in polytherapy. Some new ASMs have specific contraindications, such as LCM, rufinamide, and retigabine in patients with long QT syndrome, as well as TPM, zonisamide, and LEV in patients with neuropsychiatric comorbidities because of increased risk of anxiety, depression, and psychosis (44). It is interesting to highlight that the use of polytherapy does not necessarily imply an increase of AEs. According to some studies, the risk of AEs was similar between patients in monotherapy and polytherapy, and patients belonging to the second group were even able to tolerate higher total drug load (TDL, ratio of prescribed daily dose defined by WHO) compared to those in monotherapy. Authors suggested that the onset of AEs in polytherapy was not strictly related to the number of drugs but rather to the type and the dosage of the ASMs chosen and the individual susceptibility (65, 66) (see Table 1).

**Table 1:** In this table, the main ASMs combinations in several human studies are showed.

**Drug combinations**	**References**	**Efficacy**	**Potential drug-drug interaction and side effects**	**Seizure types and/or epileptic syndromes**
VPA + LTG	(51)	VPA + LTG > CBZ or PHT+ LTG	VPA-LTG combination shows supra-additive or additive effects. VPA acts as an enzymatic metabolic inhibitor, thus the addition of LTG to VPA led to a reduction in lamotrigine clearance, resulting in an increase of its plasma concentration. This combination augments the probability of lamotrigine-induced hypersensitivity and the development of both postural and action tremor. Lamotrigine should be slowly titrated with small initial dose.	Focal refractory seizures; no-specified DRE; possible combination therapy for absence seizures (67).
VPA + ETX	(52)	VPA + ETX> VPA or ETX as monotherapy	The interaction between VPA and ETX in terms of pharmacokinetic effects is not clear. It may lead to elevated serum ETX levels, even if neurotoxic effects are less than additive antiseizure effects.	Absence seizures
LTG + LEV	(53)	LTG + LEV > LEV as monotherapy	Supra-additive effect. Levetiracetam has not been reported to cause relevant pharmacokinetic drug interactions. It is not an enzyme-inducer, thus an interaction with LCM seems unlikely.	Idiopathic generalized epilepsy and post-traumatic focal epilepsy
LCM + LEV	(54, 55)	LCM + LEV > LCM + other ASMs (e.g., VPA, TPM, PB)	Supra-additive effect (may be due to different mechanism of actions). A clinically relevant interaction between LEV with LCM seems unlikely. Attention must be paid to using LCM in patients with arrhythmias or long QT syndrome and LEV in patients with neuropsychiatric disorders.	Focal onset seizures in adults.
VPA + CLB + STP	(58–60)	VPA + CLB + STP >>> VPA + CLB in duotherapy	The supra-additive effect of the STP-CLB combination may be due to pharmacokinetic drug interactions, as STP is known to act by inhibiting the metabolism of norclobazam, thus increasing its serum levels and possibly leading to more side effects (neurotoxicity effect). Common side effects are drowsiness and loss of appetite.	Dravet Syndrome

*The comparison of efficacy between different associations is simplified by major or minor symbols (>; <). The combination of valproate (VPA) with lamotrigine (LTG) has been widely assessed in several studies (some of these mentioned above) and reached the best level of evidence of efficacy. Potential adverse events related to drug-drug interactions and seizure types/epileptic syndromes of the population studied are also reported. VPA (valproic acid); LTG (lamotrigine); ETX (ethosuximide); CBZ (carbamazepine); CBZ-CR (controlled-release carbamazepine); PHT (phenytoin); LEV (levetiracetam); TPM (topiramate); PB (phenobarbital); CLB (clobazam); LCM (lacosamide); STP (stiripentol)*.

Therefore, choosing an optimal ASMs regimen for a pharmacoresistant patient in a rational polytherapy needs to consider many variables not only regarding the pharmacodynamic and pharmacokinetic aspects of ASMs, but also patient-related factors such as age, compliance, comorbidities, and concomitant medications. Finally, epilepsy syndrome and seizure types should guide the physician in the choice of the best ASMs in the management of DRE.

### Antiseizure Medications in Refractory Epileptic Syndromes

Idiopathic epileptic syndromes comprise a wide variety of focal and generalized epilepsies, mostly affecting children or adolescents, that have in common a known or presumed genetic origin and a recognizable electroclinical pattern. The majority of these patients usually show spontaneous remission or achieve seizure freedom with treatment, but a subset of them (20–30%) may present ongoing seizures despite a first and second-line ASM therapy. Treatment options in pharmacoresistant childhood absence epilepsy syndrome (CAE) which does not respond to ETX, VPA, or LTG in monotherapy as first choice ASMs, could benefit from the combination of VPA and LTG (67). As reported before, this combination showed a synergistic interaction and may be substantially more effective used in duotherapy rather than either one apart. The association of VPA and ETX in CAE has been shown to be effective in children refractory to either VPA or ETX in monotherapy (52), although no more recent studies have compared VPA-ETX combination with VPA-LTG. Other ASMs that may be tried in case of failure of these options include CLB, zonisamide, and TPM; notably, PHT, CBZ, and barbiturates are ineffective and contraindicated in absence seizures (67, 68). Furthermore, well-defined developmental and epileptic encephalopathies such as DS, LGS, and early-onset epileptic encephalopathy of infancy are instead constitutively pharmacoresistant and almost always require combination therapy. In the context of developmental and epileptic encephalopathies, DS represents a prototype of pharmacoresistant epileptic syndrome (69). It is a genetic condition primarily associated with loss-of-function mutations in *SCN1A*, a gene encoding for voltage-gated sodium channels, with the resulting loss of action in gamma-aminobutyric acid (GABA)ergic interneurons. As mentioned before, STP was first approved as adjunctive therapy in DS in combination with VPA and CLB, which are the first-line drugs in the pharmacological management of these patients. The use of TPM could be considered a second-line therapy as an alternative to STP in add-on therapy with CLB and VPA (70). Besides, emerging clinical trials have demonstrated the efficacy in seizure-control of new pharmacological therapies for DS, further discussed below in the text: cannabidiol (CBD) and fenfluramine (FFA). CBD has reached class I evidence of efficacy as an ASM in DS in addition to other medications and could be used as second-line therapy with or without STP. There is promising clinical evidence for the use of FFA on low-dose in DS, but efficacy and safety are still to be confirmed (58).

LGS is one of the most severe pediatric epileptic encephalopathies, accounting for 1–10% of all childhood epilepsies and a peak of incidence between the 3rd and 5th year of age. It has a relatively heterogeneous etiology (genetic, structural, metabolic, or unknown), a specific pattern of multiple seizure types (tonic, atonic, drop attacks), EEG abnormalities, and typical refractoriness to treatment with only short remission periods (71). RCTs of monotherapy and head-to-head comparison of add-on ASMs are currently lacking for these patients. VPA is still considered the first-line therapy for the treatment of newly diagnosed LGS. LTG is often regarded as the best choice in case of failure of VPA, with the highest efficacy against drop attack seizures, while contraindicated in myoclonic seizures because of its potential worsening effect. Rufinamide, TPM, and CLB have been shown to be effective in combination therapy with a good level of evidence as well. Zonisamide, LEV, perampanel (PER), and FFA may have a possible efficacy in this syndrome, although the level of evidence is not yet consistent (71). On the other hand, CBD has obtained a good class of evidence of efficacy in LGS as in DS; hence its use should be encouraged whenever other treatments have failed (72, 73).

### A Practical Approach for the Management of Patients With Drug-Resistant Epilepsy

Generally, newly diagnosed patients with epilepsy are initially treated with monotherapy since the evidence shows that 50% of patients with untreated epilepsy achieve prolonged seizure control (>12 months) with a first drug regimen (74). According to several experts, polytherapy should be considered only after the failure of at least two or three monotherapies (75–77). Nevertheless, recent evidence suggests to choose duotherapy as an alternative to second drug monotherapy after failure of the initial monotherapy, thus obtaining seizure remission of 15–20% with about 60% of patients that may achieve seizure freedom after the second drug trial (41, 78).

Patients who failed to respond to the second drug trial, either in monotherapy or duotherapy, satisfy the ILAE criteria for DRE definition and should be referred to dedicated epilepsy centers (2). However, they should be re-assessed (i.e., with prolonged video-EEG, neuroimaging) to eventually make the correct diagnosis of epilepsy type or exclude the causes of pseudo-pharmacoresistance mentioned above in this review. Patients who may benefit from surgical treatment like those caused by focal epileptogenic lesions have a higher likelihood of seizure remission (about 60–80%) if they are promptly referred to specialized centers rather than continue subsequent drug trials. Otherwise, if the lesion cannot be completely surgically resected without consequent neurologic morbidities, systematic trials of a second duotherapy or a triple therapy may be required, with a lower percentage of remission (41, 78). Thus, selecting potential candidates for polytherapy represents a crucial step and leads to relevant practical implications in order to increase the possibility of seizure control. Nevertheless, the addition of a fourth drug should be rather avoided, as the use of more than three drugs raises the likelihood of AEs, compared to a scarce improvement of seizure control (79). If a 5th or 6th drug trial with duo-triple or quadruple therapy failed, other alternative therapies should be evaluated. Vagus nerve stimulation (VNS) or ketogenic diet may be actively pursued. If the patient has already been treated with previous ASMs, it is crucial to carefully evaluate administration doses, efficacy, and the spectrum of AEs of previously given drugs.

### Emerging Antiseizure Medications to Overcome Drug Resistant Epilepsy

Despite the development of a huge number of ASMs in the last decades, about 30% of patients with epilepsy is refractory to medical treatment and are in dire need of new treatment options to both successfully control seizures and improve quality of life (2). Due to the multifactorial genesis of DRE and the difficulty to understand how different mechanisms could interplay each other in the same patient or group of patients, the task of overcoming drug resistance with new pharmacological treatments remains still challenging.

Recently approved ASMs as add-on treatment in patients with focal seizures, with or without secondarily generalization, and primary generalized tonic-clonic seizures are PER and brivaracetam (BRV). PER is a first-in-class, non-competitive α-amino-3-hydroxyl-5-methyl-4-isoxazole-propionate (AMPA) receptor antagonist, and it acts as a potentially broad-spectrum ASM. PER efficacy in patients with drug-resistant focal epilepsy was assessed in 3 double-blind randomized placebo-controlled phase III clinical trials (RCTs 304, 305, 306) and in an open-label extension trial (study 307) (80–84). The approved use of PER for primary generalized tonic-clonic (PGTC) seizures was based on a double-blind randomized placebo-controlled trial (study 332) that provides ILAE Class I evidence of reduced seizure frequency in refractory idiopathic generalized epilepsy (study 332) (85, 86).

BRV is currently approved for adjunctive treatment in patients with focal onset seizures. Similar to levetiracetam (LEV), BRV acts by binding SV2A vesicles with a high affinity and a linear pharmacokinetic profile. In adults with drug-refractory focal epilepsy, add-on BRV has been proven to be effective to reduce seizure frequency and fairly well-tolerated. Further studies are needed to draw definitive conclusions about its efficacy in non-LEV-naive participants and evaluate its long-term safety profile (87). Increasing evidence also supports its prescription to pediatric patients thanks to its efficacy and tolerability profile (88).

Among the new molecules studied for DRE, CBD, and FFA have demonstrated good evidence of efficacy in clinical trials in DRE. In one of the first clinical trials, CBD—the non-psychoactive compound derived from cannabis—has been approved as a purified CBD oral solution in combination therapy with CLB in DS and LGS patients from the age of 2 years (72). CBD has a distinctive chemical structure and mechanism of action compared to other new ASMs and represents the first in this new class of drugs. At clinically significative concentrations, it does not show psychoactive effects, but it acts on multiple targets as an anti-seizure molecule, including antagonism of G protein-coupled receptor 55 (GPR55), desensitization of transient receptor potential of vanilloid type 1 (TRPV1) channels, and positive allosteric modulation of GABAa receptors (72, 89). A first phase II, randomized, double-blind, placebo-controlled trial of CBD was performed on children and adolescents with DS (90, 91); then two phase III, randomized, double-blind, placebo-controlled trials demonstrated CBD effectiveness and safety as an adjunctive drug in patients with LGS, especially for the control of drop attack seizures (92, 93). An open-label extension study confirmed these data (94). No dose-response correlation (10 vs. 20 mg/kg/day) was found in a subsequent meta-analysis (95), but recent evidence showed that adjunctive CBD at the dose of 10 or 20 mg/kg/day led to similar reductions in convulsive seizure frequency for both dosage of 10 and 20 mg/kg/day, with better safety and tolerability profile for the 10 mg/kg/day dose (96). Common AEs linked to CBD were vomiting, fatigue, pyrexia, decreased appetite, somnolence, lethargy, and diarrhea (72). Whether any of the therapeutical and adverse effects of CBD could be related to the association with CLB as a concomitant medication is yet to be established. Indeed, evidence suggested that CBD significantly affects levels of CLB/N-desmethylclobazam, throughout the effect on the cytochrome p450 as inhibitor enzyme (97). However, even the likelihood synergistic effect, robust data indicate that CBD is both effective with or without the association to CLB (98–100). On the other hand, the use of FFA as ASM emerged in an unusual way since it was first approved as a weight-loss drug, then withdrawn in 1997 because of cardiac effects (valvular hypertrophy and pulmonary hypertension) (101). FFA is an amphetamine derivative and exerts its antiseizure effect by disrupting vesicle storage of serotonin, inhibiting its reuptake from the synapse, and by positive modulation on sigma 1 receptor. Furthermore, its metabolite norfenfluramine shows a high affinity for serotonin receptors in the brain (especially on 5HT2C and 1D, 5HT2A not clear) (102). Thus, FFA has been continued to use, as reported in many case series, in children with different types of epilepsy, among which pharmacoresistant patients (58). A group of child neurologists in Belgium continued to use FFA in children with DS at lower doses, showing encouraging results (103). The efficacy of the use of FFA in seizure control has been analyzed in several studies involving pediatric patients with DS and LGS (104–107). In a perspective, open-label study involving patients with DS treated with FFA at the mean dose of 0.35 (0.16–0.69) mg/kg/day for a median period of 1.5 years, it resulted in a median seizure reduction of 75% (104). Two multicenter, double-blind, placebo controlled, randomized clinical trials including children with DS treated with STP-inclusive FFA drug regimens (with variable dose from 0.2 to 0.7 mg/kg/day, maximum 30 mg/die), showed a greater response rate in term of reduction in convulsive seizures in the group treated with FFA compared to placebo, with no cardiac side effects observed (105, 106). The lack of cardiac AEs resulted from lower doses used as an ASM (20–30 mg/die maximum) compared to weight loss drug doses (up to 60 mg/day) (108). The most common non-cardiovascular AEs were anorexia, diarrhea, nasopharyngitis, lethargy, somnolence, and pyrexia. The same results of efficacy, safety, and tolerability, even if less consistent in terms of the number of clinical trials performed, were achieved for the use of FFA in LGS at different dose regimens (from 0.1 to 0.8 mg/kg/day). Randomized controlled trials are still ongoing; thus in the next future, we will be able to confirm these results (102, 107).

Besides CBD and FFA, there are emerging promising studies on the use of cenobamate and padsevonil as well, widely discussed by Loscher et al. (11). According to the “target hypothesis” mentioned above, one of the current approaches in the research of new effective molecules for DRE is the development of more effective ASM by revised target-based drug discovery. In this scenario, it has been designed padsevonil, a compound with a mechanism of action similar to LEV and brivaracetam, but with a higher affinity on presynaptic SV2 proteins and a broader effect on different SV2 subtypes. Currently, padsevonil is undergoing a phase III trial in patients with multidrug-resistant focal seizures (109). Cenobamate has been studied with the same rationale, and it is a new ASM recently approved for the treatment of partial-onset seizures in adults, which works enhancing inhibitory currents through GABAa receptor modulation and decreasing excitatory sodium current (110).

Another interesting approach at the age of genomic is “precision medicine.” The advent of genomic technologies now allows to characterize the genetic background of epilepsy better, and it is slowly changing the way to classify epileptic syndromes. Different patterns of gene mutations could underlie the same epileptic syndrome and be responsible for different drug-response, and mutations of the same gene may result in different phenotypes (11). The list of genes carrying rare pathogenic variants is growing rapidly. These discoveries have led to rational treatment strategies, including a better selection of ASMs from those existing or repurposing drugs previously not used for epilepsy.

Indeed, in some cases, it is possible to correct specific metabolic defects (e.g., the ketogenic diet for GLUT1 deficiency, or pyridoxine for pyridoxine-dependent epilepsies) (111), avoid ASMs that can potentially aggravate the pathogenic defect (e.g., the administration of sodium channel blocking drugs in SCN1A-related DS), contrast the functional defect caused by gene mutation using existing ASMs (e.g., using sodium channel blockers in SCN8A or SCN2A pathogenetic variants with gain-of-function effect), or using medications already available in the market for other indications (e.g., memantine used to treat epileptic encephalopathy caused by GRIN2A mutation of NMDA glutamate receptors) (112). Nonetheless, the only two validated examples of this precision medicine are the use of everolimus in Tuberous Sclerosis Complex-associated focal epilepsy and the use of CBD and FFA in DS, while most of the reported interesting gene-specific treatments are only based on case reports or short-term studies (113).

## Alternatives to Pharmacological Treatment in Drug-Resistant Epilepsy

The best and potentially resolutive alternative to ASMs for patients with refractory epilepsy is surgical treatment, whenever it is possible. In some cases delaying surgery may worsen the post-surgical chances to achieve seizure freedom; therefore, it is crucial to promptly identify patients who are potential candidates for intervention, as discussed above. Focal lesions commonly resected in focal epilepsy include hippocampal sclerosis and focal cortical dysplasia, with a greater success shown in patients with MRI lesions that are concordant to clinical seizures (114, 115). However, in a significant number of patients, the epileptogenic zone cannot be identified or surgically treated because of its localization within functional brain tissue. For this group of patients, neurostimulation is becoming an increasingly accepted alternative or complementary treatment to pharmaceuticals (116). Nowadays, several neurostimulation modalities are available for DRE, either invasive, requiring a surgical procedure to implant the device, or non-invasive, with no permanent device implantation required. Some devices provide continuous stimulation (open loop), whereas others deliver stimulation based upon detected brain activity (closed-loop). Among invasive methods, the most studied and established neurostimulation approach is VNS, followed by deep brain stimulation (DBS), the more recent responsive neurostimulation (RNS), and chronic subthreshold cortical stimulation (CSCS). Non-invasive alternatives are transcutaneous vagus nerve stimulation (tVNS), trigeminal nerve stimulation (TNS), transcranial magnetic stimulation (TMS), and transcranial direct current stimulation (tDCS) (117, 118). Unfortunately, despite the growing number of devices for neurostimulation, there is relatively poor knowledge about the underlying mechanisms and a lack of guidelines or general consensus across epilepsy centers on when and how to use these treatments.

VNS has been approved in the USA by Food and Drug Administration (FDA) as an adjunctive treatment for DRE in 1997, but its use has also been studied in other fields of medicine (e.g., depression, heart failure, stroke, and tinnitus) (119). Although initially approved for partial-onset seizures in people aged over 12 years, VNS is now used in adults and children with DRE not eligible for resective surgery, suffering from either focal or generalized seizures (120). This approach consists of periodic electrical stimulation delivered to the vagus nerve supplied by a programmable pulse generator, generally implanted subcutaneously under the left clavicle and connected to a lead wire wrapped around the vagus nerve distal to the recurrent laryngeal nerve. Stimulation of the left vagus nerve is preferred because it is thought to have less likely cardiac effects (e.g., bradycardia) rather than the right nerve that innervates the sinoatrial node directly (119). The exact mechanism of action of VNS on seizure control is not yet fully understood. It possibly involves first the nucleus tractus solitarius, which consequently projects to other crucial regions such as locus coeruleus, raphe nuclei, thalamus, hypothalamus, and limbic circuit. Therefore, modulation of noradrenergic and serotoninergic projections seems to be relevant, as demonstrated by increased levels of serotonin or its precursor in patients treated with VNS. Concording with the antiseizure effect, other amino acid changes in CNS reported after VNS are increased levels of the inhibitory neurotransmitter GABA and decreased levels of excitatory amino acid aspartate (121). An altered function of the limbic system, reticular activating system, and cortical structures (i.e., orbitofrontal cortex, temporal lobes) showed by functional neuroimaging, and the even stronger evidence of desynchronising effect on EEG scalp registration of VNS are thought to contribute to the antiseizure effect of this treatment (119). Traditional VNS includes baseline stimulation (open-loop) and magnet mode (on-demand). Baseline stimulation is the primary operating paradigm in which the device continually cycles with an intermittent stimulation active 24 h per day with on and off periods (e.g., 30 s on and 5 min off). Magnet mode stimulation allows the patient or caregiver to deliver adjunctive stimulation at the occurrence of a seizure, triggered by swiping a magnet over the pulse generator (122). Responsive VNS is a modern approach based on closed-loop auto-stimulation, which automatically delivers stimulation triggered by ictal tachycardia, a marker for seizure onset occurring in >80% of both generalized and focal seizures (119, 122). Generally, it is recommended to start with baseline stimulation 2 weeks after device implantation. In the setting of the device, stimulation parameters to be modulated include pulse width (starting with 250–500 μs), frequency of pulses (starting from 20 to 30 Hz), and duration of on/off times (generally 30 s on and 5 min off). Output current is initially set to 0.25 mA and gradually increased to therapeutic levels (1.25–2.0 mA), depending on patient clinical response and tolerability. Evidence from main RCTs performed on drug-resistant patients treated with VNS showed a responder rate (at least 50% reduction in seizure frequency) of 26–40% (123). The high stimulation paradigm resulted significantly better in reducing seizure rate compared to low frequency VNS (risk ratio 95% CI ranging from 1.13 to 2.64) (124). Only one RCT was conducted in children and showed no statistically significant difference in seizure frequency reduction between high and low stimulations (125). VNS is well-tolerated, with no significant differences in terms of AEs between the two stimulation paradigms. The main AEs reported are hoarseness, cough, dyspnea, pain, paresthesia, nausea, and headache (124). High-quality evidence from RCTs focused on focal epilepsies in adult patients with DRE, and however observational studies suggest VNS may also be effective in generalized epilepsies (119).

DBS is an invasive non-pharmacological treatment approved by FDA in 2018 for DRE in adults (>18 years) when surgical resection is contraindicated. A predetermined (open-loop) electrical stimulation to deep brain structures such as the anterior nucleus of the thalamus (ANT), hippocampus (HC), the centromedian nucleus of the thalamus (CMT), cerebellum, and globus pallidus, is delivered throughout implanted electrodes connected to a pulse generator. Although the mechanism is not completely understood, DBS is thought to disrupt networks responsible for seizure propagation in which thalamic activity is involved, resulting in reducing interictal discharges (118). In the SANTE (Stimulation of the Anterior Nucleus of the Thalamus for Epilepsy) trial, 110 adult patients with localization-related epilepsy were enrolled and showed a median seizure rate reduction of 40% after the 3-month blinded phase and of 69% after 5 years of follow up (126, 127). According to a recent review, stimulation of the anterior nucleus of the thalamus (ANT) and hippocampus (HC) has been shown to decrease the frequency of refractory seizures, with half of all patients enrolled in clinical studies experimenting 46–90% seizure reduction for ANT-DBS and 48–95% with HC-DBS (128). On the contrary, less evidence of efficacy is available for the stimulation of other targets, and no RCT involved pediatric patients (118, 128). Even if generally well-tolerated, the most frequent AEs reported in the SANTE trial were pain in the implant site and paresthesias (126). A higher rate of depression and memory impairment was detected in the active group compared to sham stimulation, even if not confirmed at the 5 years follow-up study (127). Noteworthy, certain seizure types and syndromes appear more sensible to the stimulation of specific targets, such as HC stimulation for temporal epilepsies and CMT stimulation for LGS and generalized seizures (129, 130).

Another neurostimulation approach for DRE is RNS, which is based on a closed-loop device capable of detecting specific patterns of epileptogenic activity and delivering focal stimulation to abort seizure activity. It consists of a pulse generator implanted under the scalp, a depth lead placed through stereotactic software in the ictal onset zone or a subdural lead implanted through a burr hole and positioned on the desired cortical area, and an external programmer by which it is possible to modulate detection and stimulation parameters upon patient characteristics (131). Main RNS indications are the treatment of adult patients with focal DRE, who have focal-onset seizures with no more than two epileptogenic foci and who have three or more disabling seizures per month. In addition to seizure frequency reduction, RNS allows long-term electrocorticography, useful for tracking seizure detections over time or determining the exact laterality of seizure onset (118, 131). An RCT showed a median seizure reduction of 53% in 256 adult patients 2 years after implantation (132). As demonstrated in other neurostimulation modalities (e.g., DBS), both percentages of seizure reduction and responder rate continued to improve over time in follow-up studies (133–135). No severe AEs were reported, but those typical of other neurostimulation devices, whereas the improved quality of life and cognitive domains were long-term registered (134). On the contrary, there is no adequate evidence for RNS use in pediatric age.

Similar to VNS, chronic subthreshold cortical stimulation (CSCS) targets the ictal cortical focus area but with a continuous subthreshold stimulation (open-loop) (136). However, few data on clinical data are now available and only based on retrospective studies. In addition to the invasive neurostimulation modalities described above, TMS, tDCS, tVNS, and TNS are recently emerging as non-invasive alternative approaches.

In TMS an external magnetic flux applied on the scalp generates intracranial currents, which can excite action potentials and modulate specific cortical circuits, thus reducing the probability of seizure recurrence. Even if it is safe (the most AE reported is migraine after stimulation) and used in the treatment of several conditions such as depression, migraine, pain, or movement disorders, evidence of efficacy in patients with DRE is of low quality due to either a relatively small and heterogeneous number of patients enrolled in the studies and non-unique results in terms of seizure reduction (137, 138).

As for TMS, the use of tDCS has been validated for many neurological and psychiatric diseases (i.e., Parkinson, pain, depression, motor function, and cognition) and epilepsy, as well. tDCS delivers direct current to targeted cortical regions through the application of scalp electrodes (anode and cathode), which can decrease (cathodal) cortical excitability, suppress seizures, and interfere with epileptiform discharges seen on EEG (139). According to different studies, there is moderate to very-low quality evidence that repeated tDCS is effective in drug-resistant patients, in particular with mesial temporal lobe epilepsy, hippocampal sclerosis, and LGS (117, 139, 140). Transcutaneous VNS (tVNS) and trigeminal nerve stimulation (TNS) have been investigated in small studies. Although well-tolerated, there is low-quality evidence of efficacy for DRE treatment (117).

In summary, current data available support the efficacy and the safety of VNS, DBS, RNS, and tDCS for DRE in adult patients, mainly with focal seizure onset epilepsy. Other approaches are generally well-tolerated, but not yet supported by adequate evidence. Noteworthy, caution should be suggested when applying VNS in patients with apnea or DBS in those with depression or cognitive impairment because of a possible worsening of these symptoms (117).

Among non-pharmacological treatments for DRE there is the ketogenic diet. This strict dietary regimen has been generally reserved for a specific group of children with DRE and is characterized by high-fat and low-carbohydrate levels, which mimic fasting (141, 142). However, it is not usually long-term used due to concerns about effects on growth and overall health. Due to a lack of RCTs and the small number of patients enrolled in the studies reviewed, the evidence for the use of ketogenic diet for DRE results of low to very low quality; thus further researches are needed, especially for adults (143). Nevertheless, if well-tolerated, the ketogenic diet may remain a valid option for drug-resistant patients.

## Conclusions

The management of drug-refractory patients is definitely still a great challenge for physicians. The heterogeneity of clinical manifestations, in terms of both seizure semeiology and course, with variable and often unpredictable periods of remission and relapses in patients with DRE, makes it difficult to compare clinical studies and define guidelines. The pathogenetic theories known so far, based mainly on pre-clinical studies, do not give a unique and integrated explanation of drug resistance, but only of some mechanisms that may underlie it and could become the target of new molecules developed by research. Although not resolving and burdened by unavoidable side effects and drug-drug interactions in patients undergoing polytherapy, pharmacological treatment remains the first and main approach to achieve long-term seizure control. After a correct diagnostic classification and the selection of patients candidates for resective surgery, it is mandatory to have adequate knowledge of the drugs, with their pharmacodynamic and pharmacokinetic profile, possible adverse effects, and the most effective pharmacological associations, in order to choose adequate drug regimens tailored on the patient. In the new second, third, and last generation ASM era, rational polytherapy has acquired more relevance, thanks to the development of drugs that have different and potentially synergistic mechanisms of action, as well as better safety and efficacy profiles compared to first generation ASMs. Therefore, before considering a patient completely unresponsive to drug therapy, it is necessary to re-assess the diagnosis of epilepsy itself and, whenever available, the genetic background (as seen, in some cases, even genetics can lead to choose one type of drug rather than another) and critically consider previous drugs administered to try subsequent more adequate therapeutic regimens. Alternative non-pharmacological approaches such as electrical stimulation and diet therapy are also promising, but they are not long-term resolutive from the evidence we have so far.

## Author Contributions

AF put forward the conception of the review and wrote the manuscript. AV, SM, and EM participated in the proposal of the concept and revised the manuscript. GBD, GD, and PS proposed suggestions for revision. All authors approved the submitted version.

## Conflict of Interest

The authors declare that the research was conducted in the absence of any commercial or financial relationships that could be construed as a potential conflict of interest.

## References

[1] Picot MC Baldy-Moulinier M Daures JP Dujols P Crespel A. The prevalence of epilepsy and pharmacoresistant epilepsy in adults: a population-based study in a Western European country. Epilepsia. (2008) 49:1230–8. 10.1111/j.1528-1167.2008.01579.x 18363709

[2] Kwan P Arzimanoglou A Berg AT Brodie MJ Allen Hauser W Mathern G. Definition of drug resistant epilepsy: consensus proposal by the ad hoc task force of the ILAE commission on therapeutic strategies. Epilepsia. (2010) 51:1069–77. 10.1111/j.1528-1167.2009.02397.x 19889013

[3] Brodie MJ Barry SJE Bamagous G Norrie JD Kwan P. Patterns of treatment response in newly diagnosed epilepsy. Neurology. (2012) 78:1548–54. 10.1212/WNL.0b013e3182563b19 22573629 PMC3348850

[4] Berg AT. Understanding the delay before epilepsy surgery: who develops intractable focal epilepsy and when? CNS Spectr. (2004) 9:136–44. 10.1017/S109285290000849X 14999169

[5] Kwan P Brodie MJ. Refractory epilepsy: a progressive, intractable but preventable condition? Seizure. (2002) 11:77–84. 10.1053/seiz.2002.0593 11945093

[6] Kwan P Schachter SC Brodie MJ. Drug-resistant epilepsy. N Engl J Med. (2011) 365:919–26. 10.1056/NEJMra1004418 21899452

[7] Dalic L Cook MJ. Managing drug-resistant epilepsy: challenges and solutions. Neuropsychiatr Dis Treat. (2016) 12:2605–16. 10.2147/NDT.S84852 27789949 PMC5068473

[8] Kalilani L Sun X Pelgrims B Noack-Rink M Villanueva V. The epidemiology of drug-resistant epilepsy: a systematic review and meta-analysis. Epilepsia. (2018) 59:2179–93. 10.1111/epi.14596 30426482

[9] Inizio Pati S Alexopoulos AV. Pharmacoresistant epilepsy: from pathogenesis to current and emerging therapies. Cleve Clin J Med. (2010) 77:457–67. 10.3949/ccjm.77a.09061 20601619

[10] Callaghan B Schlesinger M Rodemer W Pollard J Hesdorffer D Allen Hauser W. Remission and relapse in a drug-resistant epilepsy population followed prospectively. Epilepsia. (2011) 52:619–26. 10.1111/j.1528-1167.2010.02929.x 21269287 PMC3147304

[11] Löscher W Potschka H Sisodiya SM Vezzani A. Drug resistance in epilepsy: clinical impact, potential mechanisms, and new innovative treatment options. Pharmacol Rev. (2020) 72:606–38. 10.1124/pr.120.019539 32540959 PMC7300324

[12] Nei M Bagla R. Seizure-related injury and death. Curr Neurol Neurosci Rep. (2007) 7:335–41. 10.1007/s11910-007-0051-1 17618541

[13] Verrotti A D'Alonzo R Rinaldi VE Casciato S D'Aniello A Di Gennaro G. Childhood absence epilepsy and benign epilepsy with centro-temporal spikes: a narrative review analysis. World J Pediatr. (2017) 13:106–11. 10.1007/s12519-017-0006-9 28101769

[14] Matricardi S Farello G Operto FF Coppola G Verrotti A. What are the challenges with the pharmacological management of epilepsy in patients with Attention Deficit Hyperactivity Disorder (ADHD)? Exp Opin Pharmacother. (2020) 21:737–9. 10.1080/14656566.2020.1732351 32077772

[15] Coppola G Operto FF Matricardi S Verrotti A. Monitoring and managing depression in adolescents with epilepsy: current perspectives. Neuropsychiatr Dis Treat. (2019) 15:2773–80. 10.2147/NDT.S192714 31576132 PMC6765392

[16] Nickels KC Zaccariello MJ Hamiwka LD Wirrell EC. Cognitive and neurodevelopmental comorbidities in paediatric epilepsy. Nat Rev Neurol. (2016) 12:465–76. 10.1038/nrneurol.2016.98 27448186

[17] Keezer MR Sisodiya SM Sander JW. Comorbidities of epilepsy: current concepts and future perspectives. Lancet Neurol. (2016) 15:106–15. 10.1016/S1474-4422(15)00225-2 26549780

[18] Chen Z Brodie MJ Liew D Kwan P. Treatment outcomes in patients with newly diagnosed epilepsy treated with established and new antiepileptic drugs: a 30-year longitudinal cohort study. JAMA Neurol. (2018) 75:279–86. 10.1001/jamaneurol.2017.3949 29279892 PMC5885858

[19] Kwan P Brodie MJ. Early identification of refractory epilepsy. N Engl J Med. (2000) 342:314–9. 10.1056/NEJM200002033420503 10660394

[20] Schmidt D. How reliable is early treatment response in predicting long-term seizure outcome? Epilepsy Behav. (2007) 10:588–94. 10.1016/j.yebeh.2007.02.011 17418644

[21] Löscher W Potschka H. Drug resistance in brain diseases and the role of drug efflux transporters. Nat Rev Neurosci. (2005) 6:591–602. 10.1038/nrn1728 16025095

[22] Tang F Hartz AMS Bauer B. Drug-resistant epilepsy: multiple hypotheses, few answers. Front Neurol. (2017) 8:301. 10.3389/fneur.2017.00301 28729850 PMC5498483

[23] König J Müller F Fromm MF. Transporters and drug-drug interactions: important determinants of drug disposition and effects. Pharmacol Rev. (2013) 65:944–66. 10.1124/pr.113.007518 23686349

[24] Kwan P Brodie MJ. Potential role of drug transporters in the pathogenesis of medically intractable epilepsy. Epilepsia. (2005) 46: 224–35. 10.1111/j.0013-9580.2005.31904.x 15679503

[25] Sisodiya SM Lin WR Harding BN Squier MV Thom M. Drug resistance in epilepsy: expression of drug resistance proteins in common causes of refractory epilepsy. Brain. (2002) 125:22–31. 10.1093/brain/awf002 11834590

[26] Remy S Beck H. Molecular and cellular mechanisms of pharmacoresistance in epilepsy. Brain. (2006) 129:18–35. 10.1093/brain/awh682 16317026

[27] Remy S Urban BW Elger CE Beck H. Anticonvulsant pharmacology of voltage-gated Na+ channels in hippocampal neurons of control and chronically epileptic rats. Eur J Neurosci. (2003) 17:2648–58. 10.1046/j.1460-9568.2003.02710.x 12823472

[28] Bethmann K Fritschy JM Brandt C Löscher W. Antiepileptic drug resistant rats differ from drug responsive rats in GABA a receptor subunit expression in a model of temporal lobe epilepsy. Neurobiol Dis. (2008) 31:169–87. 10.1016/j.nbd.2008.01.005 18562204

[29] Fang M Xi ZQ Wu Y Wang XF. A new hypothesis of drug refractory epilepsy: neural network hypothesis. Med Hypotheses. (2011) 76:871–6. 10.1016/j.mehy.2011.02.039 21429675

[30] Schmidt D Löscher W. Drug resistance in epilepsy: putative neurobiologic and clinical mechanisms. Epilepsia. (2005) 46:858–77. 10.1111/j.1528-1167.2005.54904.x 15946327

[31] Rogawski MA. The intrinsic severity hypothesis of pharmacoresistance to antiepileptic drugs. Epilepsia. (2013) 2:33–40. 10.1111/epi.12182 23646969

[32] Balestrini S Sisodiya SM. Pharmacogenomics in epilepsy. Neurosci Lett. (2018) 667:27–39. 10.1016/j.neulet.2017.01.014 28082152 PMC5846849

[33] Löscher W Klotz U Zimprich F Schmidt D. The clinical impact of pharmacogenetics on the treatment of epilepsy. Epilepsia. (2009) 50:1–23. 10.1111/j.1528-1167.2008.01716.x 18627414

[34] Granata T Fusco L Matricardi S Tozzo A Janigro D Nabbout R. Inflammation in pediatric epilepsies: update on clinical features and treatment options. Epilepsy Behav. (2021) 107959. 10.1016/j.yebeh.2021.107959 33867302

[35] Marchi N Granata T Janigro D. Inflammatory pathways of seizure disorders. Trends Neurosci. (2014) 37:55–65. 10.1016/j.tins.2013.11.002 24355813 PMC3977596

[36] Matricardi S Farello G Savasta S Verrotti A. Understanding childhood neuroimmune diseases of the central nervous system. Front Pediatr. (2019) 7:511. 10.3389/fped.2019.00511 31921724 PMC6930888

[37] Terrone G Salamone A Vezzani A. Inflammation and epilepsy: preclinical findings and potential clinical translation. Curr Pharm Des. (2017) 23:5569–76. 10.2174/1381612823666170926113754 28950818

[38] Aronica E Bauer S Bozzi Y Caleo M Dingledine R Gorter JA. Neuroinflammatory targets and treatments for epilepsy validated in experimental models. Epilepsia. (2017) 58:27–38. 10.1111/epi.13783 28675563 PMC5873599

[39] Iorio R Assenza G Tombini M Colicchio G Della Marca G Benvenga A. The detection of neural autoantibodies in patients with antiepileptic-drug-resistant epilepsy predicts response to immunotherapy. Eur J Neurol. (2015) 22:70–8. 10.1111/ene.12529 25112548

[40] Deckers CL Hekster YA Keyser A Van Lier HJ Meinardi H Renier WO. Monotherapy versus polytherapy for epilepsy: a multicenter double-blind randomized study. Epilepsia. (2001) 42:1387–94. 10.1046/j.1528-1157.2001.30800.x 11879339

[41] Park KM Kim SE Lee BI. Antiepileptic drug therapy in patients with drug-resistant epilepsy. J Epilepsy Res. (2019) 9:14–26. 10.14581/jer.19002 31482053 PMC6706642

[42] Wilmshurst JM Gaillard WD Vinayan KP Tsuchida TN Plouin P Van Bogaert P. Summary of recommendations for the management of infantile seizures: task force report for the ILAE commission of pediatrics. Epilepsia. (2015) 56:1185–97. 10.1111/epi.13057 26122601

[43] Glauser T Ben-Menachem E Bourgeois B Cnaan A Guerreiro C Kälviäinen R. ILAE subcommission on AED guidelines: updated ILAE evidence review of antiepileptic drug efficacy and effectiveness as initial monotherapy for epileptic seizures and syndromes. Epilepsia. (2013) 54:551–63. 10.1111/epi.12074 23350722

[44] Verrotti A Tambucci R Di Francesco L Pavone P Iapadre G Altobelli E. The role of polytherapy in the management of epilepsy: suggestions for rational antiepileptic drug selection. Expert Rev Neurother. (2020) 20:167–73. 10.1080/14737175.2020.1707668 31855066

[45] Czuczwar SJ Borowicz KK. Polytherapy in epilepsy: the experimental evidence. Epilepsy Res. (2002) 52:15–23. 10.1016/S0920-1211(02)00181-X 12445956

[46] Lasoń W Dudra-Jastrzebska M Rejdak K Czuczwar SJ. Basic mechanisms of antiepileptic drugs and their pharmacokinetic/pharmacodynamic interactions: an update. Pharmacol Rep. (2011) 63:271–92. 10.1016/S1734-1140(11)70497-2 21602586

[47] Besag FM Berry DJ Pool F Newbery JE Subel B. Carbamazepine toxicity with lamotrigine: pharmacokinetic or pharmacodynamic interaction? Epilepsia. (1998) 39:183–7. 10.1111/j.1528-1157.1998.tb01356.x 9577998

[48] Barcs G Walker EB Elger CE Scaramelli A Stefan H Sturm Y. Oxcarbazepine placebo-controlled, dose-ranging trial in refractory partial epilepsy. Epilepsia. (2000) 41:1597–607. 10.1111/j.1499-1654.2000.001597.x 11114219

[49] Sake JK Hebert D Isojärvi J Doty P De Backer M Davies K. A pooled analysis of lacosamide clinical trial data grouped by mechanism of action of concomitant antiepileptic drugs. CNS Drugs. (2010) 24:1055–68. 10.2165/11587550-000000000-00000 21090839

[50] Margolis JM Chu BC Wang ZJ Copher R Cavazos JE. Effectiveness of antiepileptic drug combination therapy for partial-onset seizures based on mechanisms of action. JAMA Neurol. (2014) 71:985–93. 10.1001/jamaneurol.2014.808 24911669

[51] Brodie MJ Yuen AW. Lamotrigine substitution study: evidence for synergism with sodium valproate? 105 study group. Epilepsy Res. (1997) 26:423–32. 10.1016/S0920-1211(96)01007-8 9127723

[52] Rowan AJ Meijer JW De Beer-Pawlikowski N Van Der Geest P Meinardi H. Valproate-ethosuximide combination therapy for absence seizures. Arch Neurol. (1983) 40:797–802. 10.1001/archneur.1983.04050120047006 6416232

[53] Kinirons P McCarthy M Doherty CP Delanty N. Predicting drug-resistant patients who respond to add-on therapy with levetiracetam. Seizure. (2006) 15:387–92. 10.1016/j.seizure.2006.05.001 16766211

[54] Brigo F Ausserer H Tezzon F Nardone R. When one plus one makes three: the quest for rational antiepileptic polytherapy with supraadditive anticonvulsant efficacy. Epilepsy Behav. (2013) 27:439–42. 10.1016/j.yebeh.2013.03.010 23591263

[55] Chung S Ben-Menachem E Sperling MR Rosenfeld W Fountain NB Benbadis S. Examining the clinical utility of lacosamide: pooled analyses of three phase II/III clinical trials. CNS Drugs. (2010) 24:1041–54. 10.2165/11586830-000000000-00000 21090838

[56] Stephen IJ Sills GJ Brodie MJ. Lamotrigine and topiramate may be useful combination. Lancet. (1998) 351:958–59. 10.1016/S0140-6736(05)60613-7 9734949

[57] Brodie MJ. Pharmacological treatment of drug-resistant epilepsy in adults: a practical guide. Curr Neurol Neurosci Rep. (2016) 16:82. 10.1007/s11910-016-0678-x 27443649

[58] Cross JH Caraballo RH Nabbout R Vigevano F Guerrini R Lagae L. Dravet syndrome: treatment options and management of prolonged seizures. Epilepsia. (2019) 60:39–48. 10.1111/epi.16334 31904119

[59] Chiron C Marchand MC Tran A Rey E D'Athis P Vincent J. Stiripentol in severe myoclonic epilepsy in infancy: a randomised placebo-controlled syndrome-dedicated trial. STICLO study group. Lancet. (2000) 356:1638–42. 10.1016/S0140-6736(00)03157-3 11089822

[60] Guerrini R Tonnelier S D'Athis P Rey E Vincent J Pons G. Stiripentol in severe myoclonic epilepsy in infancy (SMEI): a placebo-controlled Italian trial. Epilepsia. (2002) 43:155. 10.1111/j.1528-1167.2002.tb06320.x

[61] Lee SK. Old versus new: why do we need new antiepileptic drugs? J Epilepsy Res. (2014) 4:39–44. 10.14581/jer.14010 25625087 PMC4295052

[62] Zaccara G Franciotta D Perucca E. Idiosyncratic adverse reactions to antiepileptic drugs. Epilepsia. (2007) 48:1223–44. 10.1111/j.1528-1167.2007.01041.x 17386054

[63] Aliyu H Ayo JO Ambali SF Kawu MU Aluwong T Dzenda T. Heamatobiochemical alterations induced by carbamazepine and phenytoin: mini review. Biochem Pharmacol. (2016) 5:219. 10.4172/2167-0501.1000219

[64] Kwan P Brodie MJ. Combination therapy in epilepsy: when and what to use. Drugs. (2006) 66:1817–29. 10.2165/00003495-200666140-00004 17040113

[65] Canevini MP De Sarro G Galimberti CA Gatti G Licchetta L Malerba A. Relationship between adverse effects of antiepileptic drugs, number of coprescribed drugs, and drug load in a large cohort of consecutive patients with drug-refractory epilepsy. Epilepsia. (2010) 51:797–804. 10.1111/j.1528-1167.2010.02520.x 20545754

[66] Lammers MW Hekster YA Keyser A Meinardi H Renier WO van LierH. Monotherapy or polytherapy for epilepsy revisited: a quantitative assessment. Epilepsia. (1995) 36:440–6. 10.1111/j.1528-1157.1995.tb00484.x 7614920

[67] Kessler SK McGinnis E. A practical guide to treatment of childhood absence epilepsy. Paediatr Drugs. (2019) 21:15–24. 10.1007/s40272-019-00325-x 30734897 PMC6394437

[68] Franzoni E Matricardi S Di Pisa V Capovilla G Romeo A Tozzi E. Refractory absence seizures: an Italian multicenter retrospective study. Eur J Paediatr Neurol. (2015) 19:660–4. 10.1016/j.ejpn.2015.07.008 26239083

[69] Brigo F Striano P Balagura G Belcastro V. Emerging drugs for the treatment of Dravet syndrome. Exp Opin Emerg Drugs. (2018) 23:261–269. 10.1080/14728214.2018.1552937 30482063

[70] Wirrell EC Laux L Donner E. Optimizing the diagnosis and management of Dravet syndrome: recommendations from a North American consensus panel. Pediatr Neurol. (2017) 68:18–34. 10.1016/j.pediatrneurol.2017.01.025 28284397

[71] Verrotti A Striano P Iapadre G Zagaroli L Bonanni P Coppola G. The pharmacological management of Lennox-Gastaut syndrome and critical literature review. Seizure. (2018) 63:17–25. 10.1016/j.seizure.2018.10.016 30391662

[72] Verrotti A Striano P. Novel therapeutic options for Dravet and Lennox-Gastaut syndrome. Expert Rev Neurother. (2021) 11:1–4. 10.1080/14737175.2020.1862651 33297778

[73] Brigo F Jones K Eltze C Matricardi S. Anti-seizure medications for Lennox-Gastaut syndrome. Cochrane Database Syst Rev. (2021) 4:CD003277. 10.1002/14651858.CD003277.pub4 33825230 PMC8095011

[74] Kwan P Brodie MJ. Epilepsy after the first drug fails: substitution or add-on? Seizure. (2000) 9:464–8. 10.1053/seiz.2000.0442 11034869

[75] Louis EKS. Truly “Rational” polytherapy: maximizing efficacy and minimizing drug interactions, drug load, and adverse effects. Curr Neuro Pharmacol. (2009) 7:96–105. 10.2174/157015909788848929 19949567 PMC2730011

[76] Brodie MJ. Medical therapy of epilepsy: when to initiate treatment and when to combine? J Neurol. (2005) 252:125–30. 10.1007/s00415-005-0735-x 15729515

[77] Shi JJ Whitlock JB Chimato N Vargas E Karceski SC Frank RD. Epilepsy treatment in adults and adolescents: expert opinion, 2016. Epilepsy Behav. (2017) 69:186–222. 10.1016/j.yebeh.2016.11.018 28237319

[78] Lee BI Park KM Kim SE Heo K. Clinical opinion: earlier employment of polytherapy in sequential pharmacotherapy of epilepsy. Epilepsy Res. (2019) 156:106–65. 10.1016/j.eplepsyres.2019.106165 31351239

[79] López González FJ Rodríguez Osorio X Gil-Nagel Rein A Carreño Martínez M Serratosa Fernández J Villanueva Haba V. Drug-resistant epilepsy: definition and treatment alternatives. Neurologia. (2015) 30:439–46. 10.1016/j.nrleng.2014.04.002 24975343

[80] French JA French JA Krauss GL Biton V Squillacote D Yang H. Adjunctive perampanel for refractory partial-onset seizures: randomized phase III study 304. Neurology. (2012) 79:589–96. 10.1212/WNL.0b013e3182635735 22843280 PMC3413761

[81] French JA Krauss GL Steinhoff BJ Squillacote D Yang H Kumar D. Evaluation of adjunctive perampanel in patients with refractory partial-onset seizures: results of randomized global phase III study 305. Epilepsia. (2013) 54:117–25. 10.1111/j.1528-1167.2012.03638.x 22905857

[82] Krauss GL Serratosa JM Villanueva V Endziniene M Hong Z French J. Randomized phase III study 306: adjunctive perampanel for refractory partial-onset seizures. Neurology. (2012) 78:1408–15. 10.1212/WNL.0b013e318254473a 22517103

[83] Krauss GL Perucca E Ben-Menachem E Kwan P Shih JJ Clément JF. Long-term safety of perampanel and seizure outcomes in refractory partial-onset seizures and secondarily generalized seizures: results from phase III extension study 307. Epilepsia. (2014) 55:1058–68. 10.1111/epi.12643 24867391 PMC4283992

[84] Krauss GL Perucca E Kwan P Ben-Menachem E Wang XF Shih JJ. Final safety, tolerability, and seizure outcomes in patients with focal epilepsy treated with adjunctive perampanel for up to 4 years in an open-label extension of phase III randomized trials: Study 307. Epilepsia. (2018) 59:866–76. 10.1111/epi.14044 29574701

[85] French JA Krauss GL Wechsler RT Wang XF DiVentura B Brandt C. Perampanel for tonic-clonic seizures in idiopathic generalized epilepsy a randomized trial. Neurology. (2015) 85:950–7. 10.1212/WNL.0000000000001930 26296511 PMC4567458

[86] Operto FF Pastorino GMG Mazza R Di Bonaventura C Matricardi S Verrotti A. Perampanel tolerability in children and adolescents with focal epilepsy: effects on behavior and executive functions. Epilepsy Behav. (2020) 103:106879. 10.1016/j.yebeh.2019.106879 31937512

[87] Lattanzi S Cagnetti C Foschi N Provinciali L Silvestrini M. Brivaracetam add-on for refractory focal epilepsy: a systematic review and meta-analysis. Neurology. (2016) 86:1344–52. 10.1212/WNL.0000000000002545 26944275

[88] Verrotti A Grasso EA Cacciatore M Matricardi S Striano P. Potential role of brivaracetam in pediatric epilepsy. Acta Neurol Scand. (2021) 143:19–26. 10.1111/ane.13347 32966640

[89] Contin M Mohamed S Santucci M Lodi MAM Russo E Mecarelli O. Cannabidiol in Pharmacoresistant Epilepsy: clinical pharmacokinetic data from an expanded access program. Front Pharmacol. (2021) 12:637801. 10.3389/fphar.2021.637801 33746760 PMC7966506

[90] Devinsky O Cross JH Laux L. Cannabidiol in Dravet syndrome study group. Trial of cannabidiol for drug-resistant seizures in the Dravet syndrome. N Engl J Med. (2017) 376:2011–20. 10.1056/NEJMoa1611618 28538134

[91] Devinsky O Patel AD Thiele EA Wong MH Appleton R Harden CL. GWPCARE1 part a study group: randomized, dose ranging safety trial of cannabidiol in Dravet syndrome. Neurology. (2018) 90:1204–11. 10.1212/WNL.0000000000005254 PMC5890607 29540584

[92] Devinsky O Patel AD Cross JH Villanueva V Wirrell EC Privitera M. GWPCARE3 study group: effect of cannabidiol on drop seizures in the Lennox-Gastaut syndrome. N Engl J Med. (2018) 378:1888–97. 10.1056/NEJMoa1714631 29768152

[93] Thiele EA Marsh ED French JA Mazurkiewicz-Beldzinska M Benbadis SR Joshi C. Cannabidiol in patients with seizures associated with Lennox-Gastautsyndrome (GWPCARE4): a randomised, double-blind, placebo-controlled phase 3 trial. Lancet. (2018) 391:1085–96. 10.1016/S0140-6736(18)30136-3 29395273

[94] Devinsky O Nabbout R Miller I Laux L Zolnowska M Wright S. Long-term cannabidiol treatment in patients with Dravet syndrome: an open-label extension trial. Epilepsia. (2019) 60:294–302. 10.1111/epi.14628 30582156 PMC7379690

[95] Lattanzi S Trinka E Russo E Striano P Citraro R Silvestrini M. Cannabidiol as adjunctive treatment of seizures associated with Lennox-Gastaut syndrome and Dravet syndrome. Drugs Today. (2019) 55:177–96. 10.1358/dot.2019.55.3.2909248 30938373

[96] Miller I Scheffer IE Gunning B Sanchez-Carpintero R Gil-Nagel A Perry MS. Dose-ranging effect of adjunctive oral cannabidiol vs placebo on convulsive seizure frequency in Dravet Syndrome: a randomized clinical trial. JAMA Neurol. (2020) 77:613–21. 10.1001/jamaneurol.2020.0073 32119035 PMC7052786

[97] Geffrey AL Pollack SF Bruno PL Thiele EA. Drug-drug interaction between clobazam and cannabidiol in children with refractory epilepsy. Epilepsia. (2015) 56:1246–51. 10.1111/epi.13060 26114620

[98] Devinsky O Thiele EA Wright S Checketts D Morrison G Dunayevich E. Cannabidiol efficacy independent of clobazam: meta-analysis of four randomized controlled trials. Acta Neurol Scand. (2020) 142:531–40. 10.1111/ane.13305 32592183 PMC7689899

[99] Anderson LL Absalom NL Abelev SV Low IK Doohan PT Martin LJ. Coadministered cannabidiol and clobazam: preclinical evidence for both pharmacodynamic and pharmacokinetic interactions. Epilepsia. (2019) 60:2224–34. 10.1111/epi.16355 31625159 PMC6900043

[100] Lattanzi S Trinka E Striano P Zaccara G Del Giovane C Nardone R. Cannabidiol efficacy and clobazam status: a systematic review and meta-analysis. Epilepsia. (2020) 61:1090–8. 10.1111/epi.16546 32452532

[101] Schoonjans AS Lagae L Ceulemans B. Low-dose fenfluramine in the treatment of neurologic disorders: experience in Dravet syndrome. Ther Adv Neurol Disord. (2015) 8:328–38. 10.1177/1756285615607726 26600876 PMC4643872

[102] Balagura G Cacciatore M Grasso EA Striano P Verrotti A. Fenfluramine for the treatment of Dravet syndrome and lennox-gastaut syndrome. CNS Drugs. (2020) 34:1001–7. 10.1007/s40263-020-00755-z 32875491

[103] Ceulemans B Boel M Leyssens K Van Rossem C Neels P Jorens PG. Successful use of fenfluramine as an add-on treatment for Dravet syndrome. Epilepsia. (2012) 53:1131–9. 10.1111/j.1528-1167.2012.03495.x 22554283

[104] Schoonjans A Paelinck BP Marchau F Gunning B Gammaitoni A Galer BS. Low-dose fenfluramine significantly reduces seizure frequency in Dravet syndrome: a prospective study of a new cohort of patients. Eur J Neurol. (2017) 24:309–14. 10.1111/ene.13195 27790834 PMC5298030

[105] Lagae L Sullivan J Knupp K Laux L Polster T Nikanorova M. Fenfluramine hydrochloride for the treatment of seizures in Dravet syndrome: a randomised, double-blind, placebo-controlled trial. Lancet. (2020) 394:2243–54. 10.1016/S0140-6736(19)32500-0 31862249

[106] Nabbout R Mistry A Zuberi S Villeneuve N Gil-Nagel A Sanchez-Carpintero R. Fenfluramine for treatment-resistant seizures in patients with Dravet syndrome receiving stiripentol-inclusive regimens: a randomized clinical trial. JAMA Neurol. (2019) 77:300–8. 10.1001/jamaneurol.2019.4113 31790543 PMC6902175

[107] Lagae L Schoonjans AS Gammaitoni AR Galer BS Ceulemans B. A pilot, open-label study of the effectiveness and tolerability of low-dose ZX008 (fenfluramine HCl) in Lennox-Gastaut syndrome. Epilepsia. (2018) 59:1881–8. 10.1111/epi.14540 30146701

[108] Lai WW Galer BS Wong PC Farfel G Pringsheim M Keane MG. Cardiovascular safety of fenfluramine in the treatment of Dravet syndrome: analysis of an ongoing long-term open-label safety extension study. Epilepsia. (2020) 61:2386–95. 10.1111/epi.16638 32809271 PMC7754414

[109] Bialer M Johannessen SI Koepp MJ Levy RH Perucca E Tomson T. Progress report on new antiepileptic drugs: a summary of the Fourteenth Eilat Conference on New Antiepileptic Drugs and Devices (EILAT XIV). I. drugs in preclinical and early clinical development. Epilepsia. (2018) 59:1811–41. 10.1111/epi.14557 30368792

[110] Krauss GL Klein P Brandt C Lee SK Milanov I Milovanovic M. Safety and efficacy of adjunctive cenobamate (YKP3089) in patients with uncontrolled focal seizures: a multicentre, double-blind, randomised, placebo-controlled, dose-response trial. Lancet Neurol. (2020) 19:38–48. 10.1016/S1474-4422(19)30399-0 31734103

[111] Ragona F Matricardi S Castellotti B Patrini M Freri E Binelli S. Refractory absence epilepsy and glut1 deficiency syndrome: a new case report and literature review. Neuropediatrics. (2014) 45:328–32. 10.1055/s-0034-1378130 24892788

[112] Perucca P Perucca E. Identifying mutations in epilepsy genes: Impact on treatment selection. Epilepsy Res. (2019) 152:18–30. 10.1016/j.eplepsyres.2019.03.001 30870728

[113] Wang J Lin ZJ Liu L Xu HQ Shi YW Yi YH. Epilepsy-associated genes. Seizure. (2017) 44:11–20. 10.1016/j.seizure.2016.11.030 28007376

[114] Simasathien T Vadera S Najm I Gupta A Bingama W Jehi L. Improved outcomes with earlier surgery for intractable frontal lobe epilepsy. Ann Neurol. (2013) 73:646–54. 10.1002/ana.23862 23494550

[115] Barba C Cross JH Braun K Cossu M Klotz KA De Masi S. Trends in pediatric epilepsy surgery in Europe between 2008 and 2015: country-, center-, and age-specific variation. Epilepsia. (2020) 61:216–27. 10.1111/epi.16414 31876960

[116] Lattanzi S Cagnetti C Matricardi S Silvestrini M. Palliative non-resective surgery for drug-resistant epilepsy. Brain Dev. (2018) 40:512–3. 10.1016/j.braindev.2017.12.012 29342410

[117] Boon P De Cock E Mertens A Trinka E. Neurostimulation for drug-resistant epilepsy: a systematic review of clinical evidence for efficacy, safety, contraindications and predictors for response. Curr Opin Neurol. (2018) 31:198–210. 10.1097/WCO.0000000000000534 29493559

[118] Starnes K Miller K Wong-Kisiel L Lundstrom BN. A review of neurostimulation for epilepsy in pediatrics. Brain Sci. (2019) 9:283. 10.3390/brainsci9100283 PMC6826633 31635298

[119] Pérez-Carbonell L Faulkner H Higgins S Koutroumanidis M Leschziner G. Vagus nerve stimulation for drug-resistant epilepsy. Pract Neurol. (2020) 20:189–98. 10.1136/practneurol-2019-002210 31892545

[120] Morris GL Gloss D Buchhalter J Mack KJ Nickels K Harden C. Evidence-based guideline update: vagus nerve stimulation for the treatment of epilepsy: report of the Guideline Development Subcommittee of the American Academy of Neurology. Neurology. (2013) 81:1453–9. 10.1212/WNL.0b013e3182a393d1 23986299 PMC3806910

[121] Ben-Menachem E Hamberger A Hedner T Hammond EJ Uthman BM Slater J. Effects of vagus nerve stimulation on amino acids and other metabolites in the CSF of patients with partial seizures. Epilepsy Res. (1995) 20:221–7. 10.1016/0920-1211(94)00083-9 7796794

[122] Fisher B DesMarteau JA Koontz EH Wilks SJ Melamed SE. Responsive vagus nerve stimulation for drug resistant epilepsy: a review of new features and practical guidance for advanced practice providers. Front Neurol. (2021) 11:610379. 10.3389/fneur.2020.610379 33584511 PMC7874068

[123] Chambers A Bowen JM. Electrical stimulation for drug-resistant epilepsy: an evidence-based analysis. Ont Health Technol Assess Ser. (2013) 13:1–37. 24228081 PMC3817921

[124] Panebianco M Rigby A Weston J Marson AG. Vagus nerve stimulation for partial seizures. Cochrane Database Syst Rev. (2015) 2015:CD002896. 10.1002/14651858.CD002896.pub2 PMC7138043 25835947

[125] Klinkenberg S Aalbers MW Vles JS Cornips EM Rijkers K Leenen L. Vagus nerve stimulation in children with intractable epilepsy: a randomized controlled trial. Dev Med Child Neurol. (2012) 54:855–61. 10.1111/j.1469-8749.2012.04305.x 22540141

[126] Fisher R Salanova V Witt T Worth R Henry T Gross R. Electrical stimulation of the anterior nucleus of thalamus for treatment of refractory epilepsy. Epilepsia. (2010) 51:899–908. 10.1111/j.1528-1167.2010.02536.x 20331461

[127] Salanova V Witt T Worth R Henry TR Gross RE Nazzaro JM. Long-term efficacy and safety of thalamic stimulation for drug-resistant partial epilepsy. Neurology. (2015) 84:1017–25. 10.1212/WNL.0000000000001334 25663221 PMC4352097

[128] Li MCH Cook MJ. Deep brain stimulation for drug-resistant epilepsy. Epilepsia. (2018) 59:273–90. 10.1111/epi.13964 29218702

[129] Lim S-N Lee C-Y Lee S-T Tu PH Chang BL Lee CH. Low and high frequency hippocampal stimulation for drug-resistant mesial temporal lobe epi-lepsy. Neuromodulation. (2016) 19:365–72. 10.1111/ner.12435 27072376 PMC5074270

[130] Valentin A Garcia Navarrete E Chelvarajah R Torres C Navas M Vico L. Deep brain stimulation of the centromedian thalamic nucleus for the treatment of generalized and frontal epilepsies. Epilepsia. (2013) 54:1823–33. 10.1111/epi.12352 24032641

[131] Matias CM Sharan A Wu C. Responsive neurostimulation for the treatment of epilepsy. Neurosurg Clin N Am. (2019) 30:231–42. 10.1016/j.nec.2018.12.006 30898274

[132] Bergey GK Morrell MJ Mizrahi EM Goldman A King-Stephens D Nair D. Long-term treatment with responsive brain stimulation in adults with refractory partial seizures. Neurology. (2015) 84:810–7. 10.1212/WNL.0000000000001280 25616485 PMC4339127

[133] Geller EB Skarpaas TL Gross RE Goodman RR Barkley GL Bazil CW. Brain responsive neurostimulation in patients with medically intractable mesial temporal lobe epilepsy. Epilepsia. (2017) 58:994–1004. 10.1111/epi.13740 28398014

[134] Nair DR Laxer KD Weber PB Murro AM Park YD Barkley GL. Nine-year prospective efficacy and safety of brain-responsive neurostimulation for focal epilepsy. Neurology. (2020) 95:e1244–56. 10.1212/WNL.0000000000010154 32690786 PMC7538230

[135] Heck CN King-Stephens D Massey AD Nair DR Jobst BC Barkley GL. Two year seizure reduction in adults with medically intractable partial onset epilepsy treated with responsive neurostimulation: final results of the RNS system pivotal trial. Epilepsia. (2014) 55:432–41. 10.1111/epi.12534 24621228 PMC4233950

[136] Lundstrom BN Worrell GA Stead M Van Gompel JJ. Chronic subthreshold cortical stimulation: a therapeutic and potentially restorative therapy for focal epilepsy. Expert Rev Neurother. (2017) 17:661–6. 10.1080/14737175.2017.1331129 28532252

[137] Lefaucheur JP André-Obadia N Antal A Ayache SS Baeken C Benninger DH. Evidence-based guidelines on the therapeutic use of repetitive transcranial magnetic stimulation (rTMS). Clin Neurophysiol. (2014) 125:2150–206. 10.1016/j.clinph.2014.05.021 25034472

[138] Lefaucheur JP Aleman A Baeken C Benninger DH Brunelin J Di Lazzaro V. Evidence-based guidelines on the therapeutic use of repetitive transcranial magnetic stimulation (rTMS): an update (2014-2018). Clin Neurophysiol. (2020) 131:474–528. 10.1016/j.clinph.2020.02.003 31901449

[139] Fregni F El-Hagrassy MM Pacheco-Barrios K Carvalho S Leite J Simis M. Evidence-based guidelines and secondary meta-analysis for the use of transcranial direct current stimulation (tDCS) in neurological and psychiatric disorders. Int J Neuropsychopharmacol. (2020) 26:pyaa051. 10.1093/ijnp/pyaa051 PMC8059493 32710772

[140] Yang D Wang Q Xu C Fang F Fan J Li L. Transcranial direct current stimulation reduces seizure frequency in patients with refractory focal epilepsy: a randomized, double-blind, sham-controlled, and three-arm parallel multicenter study. Brain Stimul. (2020) 13:109–16. 10.1016/j.brs.2019.09.006 31606448

[141] Verrotti A Iapadre G Di Francesco L Zagaroli L Farello G. Diet in the treatment of epilepsy: what we know so far. Nutrients. (2020) 12:2645. 10.3390/nu12092645 32872661 PMC7551815

[142] Operto FF Matricardi S Pastorino GMG Verrotti A Coppola G. The ketogenic diet for the treatment of mood disorders in comorbidity with epilepsy in children and adolescents. Front Pharmacol. (2020) 11:578396. 10.3389/fphar.2020.578396 33381032 PMC7768824

[143] Martin-McGill KJ Bresnahan R Levy RG Cooper PN. Ketogenic diets for drug-resistant epilepsy. Cochrane Database Syst Rev. (2020) 6:CD001903. 10.1002/14651858.CD001903.pub5 32588435 PMC7387249

